
JOURNAL INFORMATION
==============================
NLM Title Abbreviation: ScientificWorldJournal
Journal ID: ScientificWorldJournal
NLM Journal ID: 101131163
Journal ID: TSWJ

The Scientific World Journal

ISSN: 2356-6140
EISSN: 1537-744X
Publisher: Wiley

ARTICLE INFORMATION
==============================
PMCID: PMC6084129
PMID: 30147602
DOI: 10.1100/tsw.2001.221
Article ID: 717128
Article version: 1
Subjects: Short Report

Regulation of Cell Proliferation: Role of A Chromosome-Matrix Protein

Fragoso Miryam A. 1 2
Salas-Vega Mariana 1
Fernandez Viviana 2
Tahi Hassan 2
Parel Jean-Marie 2
Vega-Salas Dora E. 1
1 Dept. of Cell Biology and Anatomy, School of Medicine1, Univ. of Miami, Miami, FL 33136, USA
2 Ophthalmic Biophysics Center, Bascom Palmer Institute, Univ. of Miami, Miami, FL 33136, USA
Publication date: 2001
Electronic publication date: 2001 Jun 29
Volume: 1
First page: 121
Last page: 121
Copyright: Copyright © 2001 Miryam A. Fragoso et al.
Copyright year: 2001
License: This is an open access article distributed under the Creative Commons Attribution License, which permits unrestricted use, distribution, and reproduction in any medium, provided the original work is properly cited.
License URL: https://creativecommons.org/licenses/by/3.0/

==============================
Miami *Nature Biotechnology* Short Reports
*TheScientificWorld* (2001) 1 (S3), 121SR
ISSN 1532-2246; DOI 10.1100/tsw.2001.221

### REGULATION OF CELL PROLIFERATION: ROLE OF A CHROMOSOME-MATRIX PROTEIN

Miryam A. Fragoso[1-2], Mariana Salas-Vega[1], Viviana Fernandez[2], Hassan Tahi[2], Jean-Marie Parel[2], and Dora E. Vega-Salas[1]

Dept. of Cell Biology and Anatomy, School of Medicine[1], and Ophthalmic Biophysics Center, Bascom Palmer Institute[2], Univ. of Miami, Miami, FL 33136

**INTRODUCTION.** LFM-1 is a recently described chromosomal-matrix component cell-cycle dependent [1]. Preliminary experiments have suggested a regulatory role of LFM-1 in normal cell proliferation in human epithelia [2]. Lens Posterior Capsule Opacification (PCO) is a common and disabling health condition due to proliferation of Lens Epithelial Cells (LECs) to the posterior capsule of the lens [3] after cataract surgery. PCO is a widely studied condition with no effective medical treatment up to date. The purpose of this work is to develop an assay to control the proliferation of Lens Epithelial Cells by blocking LFM-1 protein synthesis and its potential function(s) in this mechanism.

**METHODS.** LECs were harvested from rabbit's lens capsules, dissociated and cultured under standard tissue culture conditions to be used as biological model for experiments. Phosphorothioate ODNs[4] (S-oligos, 23-mer) were designed, synthesized in the specific antisense (AS-ODNs) and random directions of the LFM-1 mRNA, and employed for treatment of cells during 4 days with replacement of fresh media/oligos every other day. Indirect immunofluorescence and western blot were performed to study the LFM-1 molecular specie(s), its rate of synthesis and sub-cellular distribution. Cell proliferation was simultaneously asserted by quantization of number of cells, and DNA cellular content measured by spectrofluorometry and video analysis as a function of the LFM-1 knocked translation.

**RESULTS AND DISCUSSION.** Cell proliferation was analyzed as a function of the expression of LFM-1 protein at the translational level. Indirect immunofluorescence images of cells treated with specific LFM-1 antisense oligonucleotides showed inhibition of LFM-1 endogenous synthesis and DNA replication with a 2n DNA content, and concomitant arrest of cell proliferation after 48hs of treatment. Approximately 70% of the cultures consisted of sparse cells, and no groups exceeding 4 cells per islet were observed with a viability rating between 90-96%. In contrast, LFM-1 protein displayed sub-cellular nuclear distribution (sparse fluorescent dots) in untreated and random controls during $G_1$ and S phases of the cell cycle suggesting a co-localization in areas of DNA replication and an involvement in the DNA synthesis machinery. No LFM-1 electrophoresis bands (87kDa precursor, 65-intermedia and 58kDa chromosome polypeptides) were detected in parallel samples treated with LFM-1 AS ODNs confirming the immunofluorescence results and supporting our previous reports in other human epithelia.

**CONCLUSIONS.** The results reported here suggest that LFM-1 plays a ubiquitous role in the control and progression of cell cycle. In fact, the blockage of the translation of the endogenous LFM-1 protein in Lens Epithelial cells resulted in the arrest of cell proliferation with a direct impact on the DNA cellular content and the progression of cell-cycle. This study suggests a role of the chromosome-scaffold in DNA replication and proliferation, and may open a new avenue to the prevention of PCO after cataract surgery.

**ACKNOWLEDGMENT.** This work was supported by a CRCERT grant in collaboration with the Ophthalmic Biophysics Center, Bascom Palmer Institute, and Sylvester Comprehensive Cancer Center, Univ. of Miami to DV-S.

**REFERENCES.**

1. Vega-Salas, D.E and Salas, P.J.I. (1996) Chromosoma 104, 321-331
2. Vega-Salas, D.E., Fernandez, III$^{rd}$, M., Stansberry, J., and Nouri, K. (submitted)
3. Parel, J.-M., Simon, G., and The Members of the Accommodation Club (1995) Phaco-Ersatz 2001: Update. An. Inst. Barraquer, (Barc.) 25, 143-151
4. Agrawal, S. (1996) Trends Biotechnol. 14, 376-387
